# Selected features of breast and peritoneal cancers diagnosed in *BRCA1* carriers after risk-reducing salpingo-oophorectomy

**DOI:** 10.1186/s13053-019-0109-5

**Published:** 2019-03-14

**Authors:** Janusz Menkiszak, Anita Chudecka-Głaz, Aneta Cymbaluk-Płoska, Aleksander Celewicz, Zbigniew Kojs, Mariusz Szajda, Maria Świniarska, Ryszard Bedner, Anna Jurczak, Marta Celewicz, Monika Cieszyńska, Jan Lubiński, Jacek Gronwald

**Affiliations:** 1 Department of Gynecological Surgery and Gynecological Oncology of Adults and Adolescents, Pomeranian Medical University, Szczecin, Poland; 2 Department of Gynecologic Oncology, Centre of Oncology, Maria Sklodowska-Curie Memorial Institute, Branch, Cracow, Poland; 3 International Hereditary Cancer Center, Department of Genetics and Pathology, Pomeranian Medical University, Szczecin, Poland; 4 Department of the Clinical Oncology the West Pomeranian Centre of the Oncology in Szczecin, Szczecin, Poland; 5 Department of Clinical Nursing, Pomeranian Medical University, Szczecin, Poland; 6 Department of Obstetrics and Gynecology, Pomeranian Medical University, Szczecin, Poland

**Keywords:** *BRCA1* mutation, Cancer, Prophylactic surgery

## Abstract

**Background:**

Since more than two decades Risk-reducing salpingo-oophorectomy (RRSO) is recommended and widely accepted by BRCA1/2 carriers as a method reducing ovarian cancer risk and improving survival rate. After RRSO, there remains a risk of breast cancer and peritoneal cancer. The characteristics of these neoplasms are not well known. In this study, we determined the selected parameters such as age at cancer diagnosis, time from RRSO to the diagnosis of cancer, and significance of *BRCA1* mutation type in patients diagnosed with breast or peritoneal cancer during postoperative follow-up.

**Methods:**

The material comprised of 195 *BRCA1* carriers who performed RRSO between years 1999–2012. In this period, 16 patients developed cancer (6-primary breast cancer, 3-contralateral breast cancer, 5-relapse of breast cancer, 2-peritoneal cancer). They were subject of the further analysis.

**Results:**

During the follow-up period mean age of patients after RRSO at the time of cancer diagnosis was 53.19. The mean age of patients diagnosed with primary breast cancer was 50, contralateral breast cancer – 58.67, recurrence of breast cancer - 51 and peritoneal cancer 60. The mean time periods from RRSO to the diagnosis of primary, contralateral, recurrence breast cancer were 53, 58.67 and 25,4 months respectively and of peritoneal cancer 46 months. *BRCA1* c.5266dupC mutation carriers demonstrated significantly shorter time of cancer development compared to patients carrying c.181T > G and c.4035delA mutations. Peritoneal cancer was only observed in two c.181T > G *BRCA1* mutation carriers.

**Conclusions:**

The mean age of cancer diagnosis and the mean time periods from RRSO to the diagnosis of cancer are similar to those observed by other researchers. The carriers of c.181T > G and c.5266dupC *BRCA1* mutation should be the subject further studies in context of breast and peritoneal cancer risk or time of cancer development after RRSO, respectively.

## Introduction

It was shown that diagnostic methods for ovarian cancer early-stage detection are ineffective [1]. Intensive screening tests in groups with the highest risk of ovarian cancer, such as carriers of the *BRCA1* and *BRCA2* mutations, have also low efficacy [1–6]. For these reasons, patients who are carriers of the *BRCA1/2* mutation are offered risk-reducing salpingo-oophorectomies (RRSO), which is widely accepted by *BRCA1/2* carriers and have been performed for decades in hospitals and gynecological clinics all around the world. At the moment, such management is considered the best possible option for reducing ovarian cancer risk and improving the survival rate [1, 7, 8]. However, after this surgery, there remains a risk of breast cancer and risk of peritoneal cancer. Data about characteristics of these cancers developing after RRSO are pure. In this study, we analyzed characteristics of breast or peritoneal cancer after RRSO. Follow-up was conducted over the course of 12 years. The analysis included: age at cancer diagnosis, time from RRSO to the diagnosis of cancer, significance of *BRCA1* mutation type in patients diagnosed with breast or peritoneal cancer during postoperative follow-up.

## Material and methods

The material comprised of 195 *BRCA1* carriers from the West Pomeranian Voivodship in Poland who performed RRSO between 15.09.1999–31.12.2012 at the Department of Gynecological Surgery and Oncology of Adults and Adolescents of the Pomeranian Medical University in Szczecin. No malignancy was found in the histopathological examination of the excised material. 80 of 195 (41.03%) patients were treated for breast cancer before risk-reducing surgery. All patients carried one of three *BRCA1* mutations most commonly occurring in the Polish population (c.5266dupC – 128 patients, c.4035delA – 19 patients and c.181T > G – 48 patients) [9]. Median follow-up time for the group of 195 patients amounted to 80 months.

At the time of observation 16 out of 195 patients were diagnosed with cancer. 6 (3.1%) cases with primary breast cancer (PBC); 3 (1.5%) cases contralateral primary breast cancer (CPBC); 5 (2.56%) cases with cancer cells diagnosed in a scar after mastectomy defined as relapse of breast cancer (RBC); 2 (1.03%) cases with peritoneal cancer. The detailed characteristics of 16 patients from the study group are shown in Table 1. Ten (62.5%) of these patients had been treated for breast cancer also before the RRSO. The median age of affected patients (*n* = 16) at the time of RRSO was 46.5 years (36–63 years), which did not deviate from median age for the whole group (*n* = 195) – 47 years (31–78 years).

**Table 1:** Characteristics of 16 patients who developed cancer after risk-reducing salpingo-oophorectomy

Patient number	BRCA1 mutation type	Cancer diagnosed before prophylactic surgery (yes/no) location	Date of prophylactic surgery (month/year)	Date of cancer diagnosis after prophylactic surgery (month/year)	Cancer diagnosed after prophylactic surgery (location)	Time from risk-reducing surgery to cancer diagnosis (months)
1.	c.5266dupC	Yes breast	02/2008	06/2008	breast - second primary	4
2.	c.5266dupC	Yes breast	06/2006	04/2008	breast -recurrence	22
3.	c.181T > G	Yes breast	09/1999	11/2004	peritoneum	62
4.	c.5266dupC	Yes breast	11/2002	05/2004	breast -recurrence	18
5.	c.181T > G	Yes breast	02/2001	08/2003	peritoneum	30
6.	c.5266dupC	Yes breast	01/2002	04/2008	breast -recurrence	75
7.	c.181T > G	no	08/2003	06/2008	breast - primary	58
8.	c.5266dupC	no	06/2002	10/2006	breast - primary	52
9.	c.5266dupC	Yes breast	09/2001	01/2002	breast -recurrence	4
10.	c.5266dupC	no	11/2001	01/2008	breast - primary	74
11.	c.5266dupC	no	04/2006	03/2008	breast - primary	23
12.	c.5266dupC	Yes breast	06/2004	06/2006	breast - second primary	24
13.	c.4035delA	no	11/2002	07/2009	breast primary	80
14.	c.181T > G	Yes breast	05/2000	09/2012	breast - second primary	148
15.	c.5266dupC	Yes breast	07/2002	03/2003	breast -recurrence	8
16.	c.5266dupC	no	11/2000	06/2003	breast - primary	31

Histopathological examination of tissues excised during RRSO was performed with extraordinary caution in order to rule out the presence of cancerous foci in the ovary or the salpinx, which have been described in the literature and could have been missed in the primary histopathological assessment [10, 11]. Repeated histopathological examination of excised material failed to reveal micro-invasive foci in both of our patients diagnosed with primary peritoneal cancer during the follow-up period.

The patient analysis included: age at cancer diagnosis, time from RRSO to the diagnosis of cancer, significance of *BRCA1* mutation type in patients diagnosed with breast or peritoneal cancer during postoperative follow-up. The data has been subjected to statistical analysis.

### Statistical analysis

All variables were checked for normal distribution using the Shapiro-Wilk test. They were described as means, standard deviations, medians, quartiles, as well as minimal and maximal values. We checked for statistically significant differences in quantitative variables using Student’s t-test or Mann-Whitney test and Fisher’s exact test were used in order to calculate differences in proportions. For all tests, the differences were considered statistically significant at *p*-value < 0.05. Statistical analyses were performed using STATA 11 software (license no. 30110532736).

## Results

Summary of selected features of *BRCA1* carriers diagnosed with cancer during the follow-up period is shown in Table 2. The mean age of cancer diagnosis in 16 patients who were subject of the study, was 53.19. Peritoneal cancer observed in two patients was diagnosed significantly later at a mean age of 60. Both patients were previously treated (before RRSO) due to breast cancer. With regards to breast cancer, the PBC was diagnosed in 6, CPBC in 3 and RBC in 5 out of 16 cases. The peritoneal cancer was diagnosed almost 7 months earlier than PBC (46 months vs. 53 months), but this difference was not statistically significant. Breast cancer recurrence was diagnosed almost twice as fast as primary breast cancer and peritoneal cancer (25.4 vs. 53 months), but this difference was also not statistically significant.

**Table 2:** Summary of selected features of *BRCA1* carriers diagnosed with cancer during the follow-up period

Type and number	Peritoneal cancer *n* = 2	Primary breast cancer *n* = 6	Contralateral breast cancer *n* = 3	Breast cancer recurrence *n* = 5	Total for the whole group diagnosed with cancers *n* = 16
Feature and type of mutation					
Patient age at the time of cancer diagnosis (mean; years old)	60	50.00	58.67	51.00	53.19
Time from RRSO to cancer diagnosis (mean; months)	46	53	58.67	25.4	44,56
Type of BRCA1 mutation in patients who developed cancer during follow-up period	2x c.181T > G	4x c.5266dupC 1x c.181T > G 1x c.4035delA	2x c.5266dupC 1x c.181T > G	5x c.5266dupC	11x c.5266dupC 4x c.181T > G 1x c.4035delA

Peritoneal cancers diagnosed during the follow-up period were observed only in two c.181T > G BRCA1 mutation carriers. We performed a statistical analysis of the frequency of peritoneal cancer in c.181T > G BRCA1 mutation carriers in comparison to other mutation carriers. Statistical significant differences were observed for this characteristic (*p* = 0.0392; OR = 29.00; 95% CI: 1.048–802.64).

The time to any cancer diagnosis after RRSO was significantly shorter for c.5266dupC mutation carriers compared to c.181T > G and c.4035delA mutation carriers (30.45 vs. 75.6 months; *p* = 0.021).

The c.5266dupC *BRCA1* mutation was most frequent among patients who developed any cancer – 68.75% (11/16), followed by c.181T > G mutation – 25% (4/16), and c.4035delA observed in 6,25% (1/16) of patients. This reflects the frequency of these mutations in the Polish population.

## Discussion

In our material, we observed relatively advanced age of cancer diagnosis among *BRCA1* carriers after RRSO. It is most likely associated with particularly late age of undergoing this surgery. In our study, the average age of undergoing such an operation is 46–49 years [12–14]. The reason for delayed RRSO was that, in a significant number of breast cancer patients, *BRCA1* mutation was diagnosed after diagnosis of breast cancer. In our study group, 62.5% of patients had been previously treated due to breast cancer. As indicated in our previous studies, patients treated for breast cancer undergo RRSO at a later age compared to patients without the diagnosis of breast cancer (50 vs. 46 years; *p* = 0.0003) [13].

In our study, we found that the mean age at diagnosis of any breast cancer was similar to those observed by Ramon et al. and equaled 52.2 vs. 51.8 years of age. The mean time from RRSO to diagnosis of breast cancer was also similar and amounted to 44,35 vs. 40.8 months [15]. Fakkert et al. reported breast cancer diagnosis among *BRCA1* patients after RRSO at a significantly younger age – 45.25 years, and somewhat longer time from RRSO to the diagnosis of breast cancer – 52.8 months [16]. Kauff et al. indicated significantly shorter time to diagnosis of breast and peritoneal cancer among patients after RRSO – 10.3 and 16.3 months, respectively [17].

Finch et al. showed that mean time to development of peritoneal cancer among *BRCA1* carriers after RRSO amounted to 63.6 months, although they emphasize that in three cases the diagnosis was made before the end of three years. The mean age of those patients at the time of diagnosis equalled 51.5 years [18], which approximates our data. In another study, Finch et al. reported similar mean age at diagnosis of peritoneal cancer - 51.6 years, but the mean time to diagnosis extended to 73.2 months. Possibly, prolonged time to the diagnosis of peritoneal cancer might have been influenced by the inclusion of four patients with *BRCA2* mutation into the study group of 32 patients [19]. In case of particularly short time to diagnosis of peritoneal cancer after RRSO, one should very carefully assess the excised material in order to rule out micro-invasive foci, which is described in the literature [10, 11].

Rhiem et al. observed a case of peritoneal cancer in a 57-year-old woman 26.4 months after RRSO [20]. Rebbeck et al. diagnosed peritoneal cancer in patients 45.6 and 103.2 months after RRSO [21]. Kiely et al. showed peritoneal cancer in a 70-year-old patient eight 96 months after RRSO [22]. In another publication, Kauff et al. reported peritoneal cancers among patients after RRSO on average after 41.16 months and breast cancers after 36.36 months [23]. Powell et al. demonstrated equally short time of diagnosis of peritoneal cancer in a patient after RRSO as Kauff [17]. The diagnosis was made as early as a year after the procedure [24]. In our material, the mean age of patients diagnosed with peritoneal cancer was 60 and the mean time from RRSO to the diagnosis of peritoneal cancer amounted to 46 months.

Influence of particular *BRCA1* gene mutation on studied characteristics is a very difficult topic to discuss. Although c.5266dupC*,* the most common mutation in the Polish population, is also often identified in the Ashkenazi Jewish population [25], available literature lacks data for discussion. The frequency of particular *BRCA1* mutations observed between carriers who were diagnosed with any cancer reflects the frequency of these mutations in the Polish population. However, we found peritoneal cancer in two *BRCA1* carriers with c.181T > G mutation, only. These two patients also developed breast cancer before RRSO. We observed that the time to any cancer diagnosis after RRSO was significantly shorter for c.5266dupC *BRCA1* mutation carriers. We think that these are interesting observations important for future analyses on significance of particular *BRCA1* gene mutation, however, for more general conclusions studies on larger groups should be performed.

## Conclusions

The mean age of cancer diagnosis and the mean time periods from RRSO to the diagnosis of cancer are similar to those observed by other researchers. The carriers of c.181T > G and c.5266dupC *BRCA1* mutation should be the subject further studies in context of breast and peritoneal cancer risk or time of cancer development after RRSO, respectively.

## Abbreviations

CI: Confidence interval
CPBC: Contralateral primary breast cancer
OR: Odds ratio
PBC: Primary breast cancer
RBC: Relapse of breast cancer
RRSO: Risk-reducing salpingo-oophorectomy
